# Recent advances in biomarkers for predicting the efficacy of immunotherapy in non-small cell lung cancer

**DOI:** 10.3389/fimmu.2025.1554871

**Published:** 2025-05-08

**Authors:** Jiacheng Zhang, Zehao Song, Yuanjie Zhang, Chentong Zhang, Qi Xue, Guochao Zhang, Fengwei Tan

**Affiliations:** ^1^ Department of Thoracic Surgery, National Cancer Center/National Clinical Research Center for Cancer/Cancer Hospital, Chinese Academy of Medical Sciences and Peking Union Medical College, Beijing, China; ^2^ Department of Thoracic Surgery, The First Affiliated Hospital of China Medical University, Shenyang, Liaoning, China

**Keywords:** NSCLC, immune checkpoint inhibitors, biomarker, tertiary lymphoid structures, circulating biomarkers

## Abstract

Lung cancer continues to be the primary cause of cancer-related deaths globally, with non-small cell lung cancer (NSCLC) accounting for approximately 85% of all instances. Recently, immune checkpoint inhibitors (ICIs) have transformed the treatment approach for NSCLC, however, only a subset of patients experiences significant benefits. Therefore, identifying reliable biomarkers to forecast the efficacy of ICIs is crucial for ensuring the safety and effectiveness of treatments, becoming a major focus of current research efforts. This review highlights the recent advances in predictive biomarkers for the efficacy of ICIs in the treatment of NSCLC, including PD-L1 expression, tertiary lymphoid structures (TLS), tumor-infiltrating lymphocytes (TILs), tumor genomic alterations, transcriptional signatures, circulating biomarkers, and the microbiome. Furthermore, it underscores the pivotal roles of liquid biopsy, sequencing technologies, and digital pathology in biomarker discovery. Special attention is given to the predictive value of TLS, circulating biomarkers, and transcriptional signatures. The review concludes that the integration of multiple biomarkers holds promise for achieving more accurate efficacy predictions and optimizing personalized immunotherapy strategies. By providing a comprehensive overview of the current progress, this review offers valuable insights into biomarker-based precision medicine for NSCLC and outlines future research directions.

## 1 Introduction

Lung cancer is a major cause of cancer-related mortality globally, with NSCLC accounting for approximately 85% of all lung cancer cases (1). In recent years, immunotherapy, particularly ICIs, has become central to treating many lung cancers, including advanced or metastatic cases. Additionally, ICIs are increasingly utilized for early-stage tumors in both neoadjuvant and adjuvant settings. The Food and Drug Administration’s (FDA’s) approval of the anti-CTLA-4 monoclonal antibody ipilimumab in 2011 marked the onset of the ICIs immunotherapy era (2). In 2015, the FDA approved the PD-1 inhibitor nivolumab for treating NSCLC after the failure of first-line platinum-based doublet chemotherapy. In 2016, pembrolizumab was authorized by the FDA as a first-line treatment for patients with high PD-L1 expression (≥50%) (3, 4). Tumor cells evade recognition by and attacks from the immune system by expressing immune checkpoint proteins. ICIs act as antibodies against immune checkpoints such as cytotoxic T-lymphocyte-associated protein 4 (CTLA-4) or programmed cell death ligand 1 (PD-L1) and programmed cell death protein 1 (PD-1), thereby restoring the ability of immune cells to kill tumor cells. However, the mechanisms of immunotherapy are extremely complex and multifactorial. The complex immune microenvironment of tumors and other characteristics of tumors also contribute to the uncertainty regarding the efficacy of ICIs treatment. Despite advancements in ICIs development for cancer treatment, the majority of patients still do not benefit from these medications. Consequently, discovering biomarkers that can predict the effectiveness of ICIs has become a primary research focus (5). Predictive biomarkers refer to biological characteristics that can predict a patient’s response to immune checkpoint inhibitor treatment, including PD-L1 expression, immune cell infiltration, tumor mutation burden (TMB), circulating markers, and microbiome composition. Identifying these markers not only aids in selecting patients who are most likely to benefit from ICIs – thereby achieving personalized treatment – but also improves the safety and effectiveness of the treatment. Furthermore, by understanding the mechanisms of these markers, researchers can develop new combination therapies to overcome resistance. Therefore, the study of predictive biomarkers predictive biomarkers not only advances the clinical application of ICIs but also lays the foundation for the future development of cancer immunotherapy, which has significant clinical and scientific importance. Additionally, screening for predictive biomarkers can reduce treatment costs for patients receiving ICIs and help optimize the allocation of medical resources.

**Figure 1:**
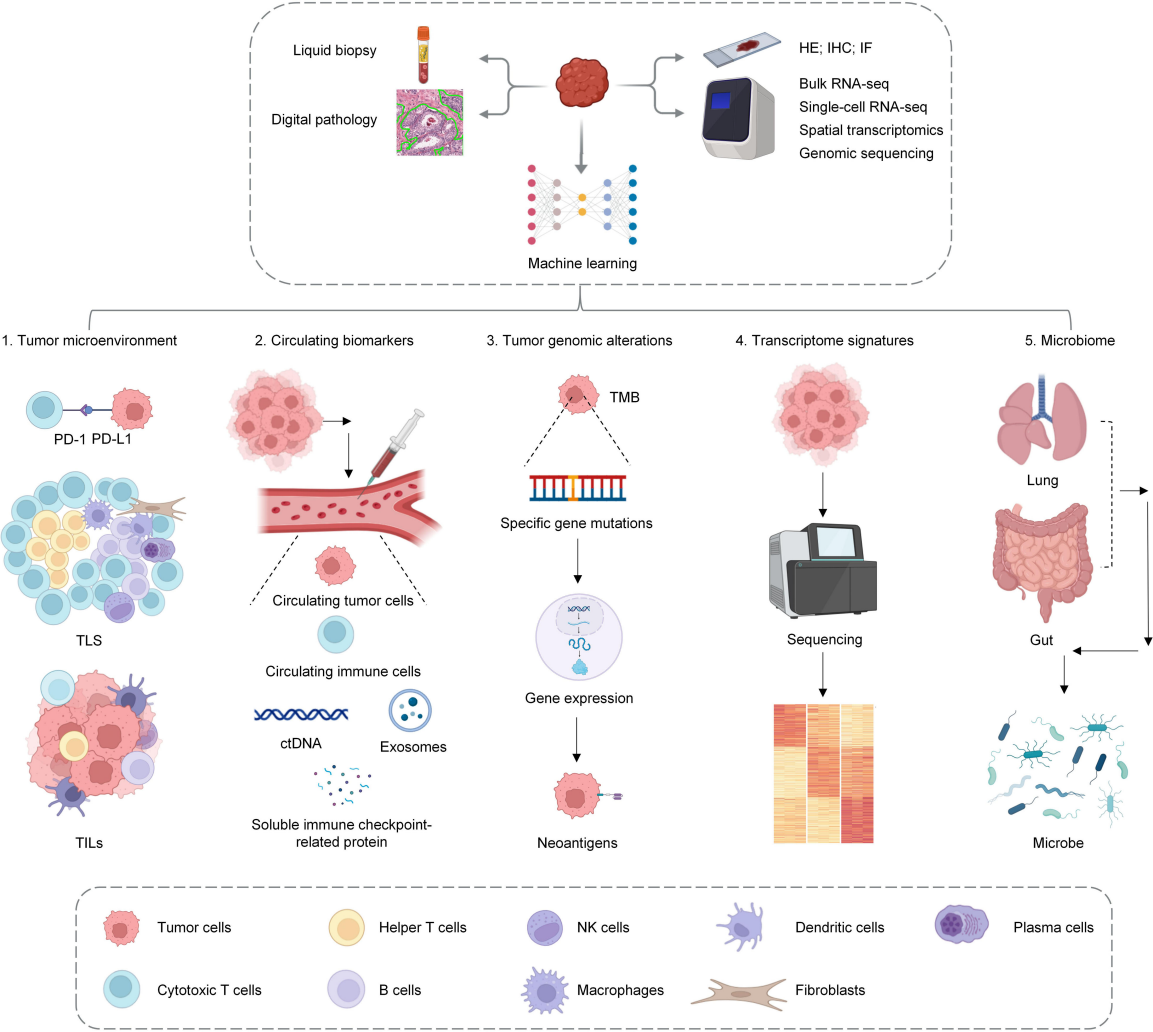
This figure serves as a summary of the main review, illustrating how various advanced techniques, including sequencing technologies, pathological analysis, liquid biopsy, and machine learning, are used to elucidate biomarkers for predicting the efficacy of ICIs. The research focuses on the analysis of the tumor microenvironment (TME), circulating biomarkers, tumor genomic alterations, transcriptional characteristics, and the microbiome.

Currently, big data is being applied more widely in the medical field; sequencing technologies are continually being popularized and optimized; the applications of digital pathology and machine learning are becoming increasingly widespread; and our understanding of various biomarkers has increased. The advancement of sequencing technologies has offered robust tools for discovering and applying biological predictive biomarkers. Genomic sequencing of tumor samples can reveal specific gene mutations, whereas transcriptomic sequencing can reflect gene expression levels. Data from epigenomics, proteomics, and metabolomics can also provide support for the discovery of biomarkers. Single-cell sequencing technology allows researchers to study the tumor microenvironment at the level of individual cells, and spatial transcriptomics preserves the spatial information of tumor tissues, thus revealing the relative positions and interactions of different cells within the microenvironment. The application of these new technologies has deepened researchers’ understanding of tumors. Digital pathology analyzes tumor tissue sections via high-resolution images, thereby allowing for a quantitative evaluation of immune cell infiltration within the TME. Machine learning algorithms can process and analyze large amounts of genomic, transcriptomic, and proteomic data to construct predictive models and improve prediction efficiency. The advancement of these new technologies has led to groundbreaking progress in the research of biological predictive biomarkers. The tumor microenvironment provides the immediate context for tumor cells and is essential in their development and progression. The interactions between lymphocytes and stromal cells that form TLSs have become a current research hotspot. TLSs are sites of immune cell accumulation and activation that provide an appropriate microenvironment to promote the maturation and activation of immune cells such as T cells and B cells. Their presence allows immune cells to quickly initiate immune responses near tumor tissue, thus significantly reducing the time required to activate the immune response. The composition, maturation status, and location of TLSs can reflect the immune characteristics of the tumor microenvironment and play crucial roles in predicting the efficacy of ICIs treatment. This review highlights the predictive role of TLSs in the treatment of NSCLC with ICIs. This review also summarizes the latest research advancements on predictive biomarkers, including PD-L1, tumor-infiltrating lymphocytes, TMB, neoantigens, transcriptomic information, circulating tumor DNA, extracellular vesicles, circulating immune cells, circulating tumor cells, specific proteins in peripheral blood, and the microbiome. We discuss the latest research on predictive biomarkers for ICIs efficacy that are under development or in the validation stage, which will play an important role in distinguishing between ICIs responders and nonresponders in the future (**Figure 1**).

## 2 PD-L1

PD-L1 is an immune checkpoint protein that suppresses T-cell activity by interacting with its receptor, PD-1. ICIs (such as PD-1 inhibitors or PD-L1 inhibitors) restore T-cell immune activity against tumors by blocking the interaction between PD-L1 and PD-1. Therefore, high PD-L1 expression may indicate tumor cell sensitivity to ICIs, and methods for detecting PD-L1 are relatively well established, thus making PD-L1 a more practical clinical predictive factor. Consequently, PD-L1 expression levels are currently the most widely accepted and commonly used biomarkers for predicting the efficacy of ICIs, regardless of smoking status (6). PD-L1 can be quantified via the TPS, TC, IC, or CPS (7). The TPS calculation method refers to the proportion of partially or completely membrane PD-L1-stained active tumor cells among all active tumor cells in the sample, and the CPS accounts for the PD-L1 expression of tumor cells and immune cells in the tumor microenvironment. Combining the TPS with the CPS may yield better predictive outcomes (8). Research that was conducted as early as 2012 indicated that a PD-L1 expression level greater than 5% was associated with the efficacy of nivolumab treatment (9). Among NSCLC patients receiving treatment with pembrolizumab, those with a PD-L1 TPS ≥ 50% had an objective response rate (ORR) of 45.2%, whereas the ORR was 16.5% for those with a TPS between 1% and 49% and only 10.7% for those with a TPS < 1% (10). Compared with those receiving chemotherapy, patients with a PD-L1 TPS ≥ 50% who received treatment with pembrolizumab had significantly improved OS and PFS. Additionally, the incidence of grade 3–5 treatment-related adverse events was also significantly lower in the pembrolizumab treatment group (11, 12). In 2016, the FDA approved pembrolizumab as a first-line or second-line treatment for NSCLC patients and indicated the role of PD-L1 expression levels in treatment (13). The CheckMate 227 trial used 1% as the cutoff for PD-L1 expression; in patients with PD-L1 expression levels ≥ 1% and PD-L1 expression levels < 1%, the 5-year survival rates for combination immunotherapy compared with chemotherapy were 24% vs. 14% and 19% vs. 7%, respectively (14). In NSCLC patients receiving CRT combined with durvalumab, those with PD-L1 expression levels ≥ 1% experienced an overall survival benefit (15). However, PD-L1 expression was temporally and spatially specific, and there may be differences between primary tumors and metastatic lesions. The optimal cutoff value for PD-L1 expression also remains unclear (16). Therefore, combining PD-L1 with other predictive indicators may provide a more accurate prediction of the efficacy of ICIs.

## 3 Tumor microenvironment

Tumors consist of both tumor and non-tumor cells, and the diverse environment created by their interactions is known as the tumor microenvironment (TME).The TME is the local environment with which tumor cells directly interact and consists of tumor cells, immune cells, stromal cells, blood vessels, and extracellular matrix components. The TME is vital in different phases of tumor development and progression (17). Furthermore, the TME can influence tumor growth and metastasis through various mechanisms, as its immunosuppressive effects potentially enable tumor cells to evade immune surveillance, thereby increasing treatment resistance. Previous studies have shown that the composition and characteristics of the TME can significantly affect the response of tumors to immunotherapy. In predicting patient survival, the quantity, density, and location of immune cells within the tumor may be more advantageous than traditional TNM staging (18). In recent years, due to advancements of high-throughput technologies, researchers have made significant progress in understanding the TME. Furthermore, the role of the TME in predicting the efficacy of ICIs has also attracted an increasing amount of attention.

### 3.1 Tumor-infiltrating lymphocytes

#### 3.1.1 CD8+ T cells

In tumor-infiltrating lymphocytes, CD8+ T cells play a crucial role as the primary effector cells in antitumor immunity. ICIs can reactivate CD8+ T cells. The efficacy of ICIs might be associated with the status of CD8+ T cells. Assessing the presence and functionality of CD8+ T cells in the TME could help identify patients who are most likely to benefit from ICIs treatment. Single-cell RNA sequencing (scRNA-seq) of NSCLC samples, both before and after anti-PD-1 treatment, showed that patients who respond to treatment have a higher number of CD8+ T cells, suggesting their potential as predictors of anti-PD-1 therapy success. Researchers have discovered two subsets of Texp cells: one with high GZMK expression and another with low expression of genes associated with exhaustion. These Texp cells significantly increase in the number of reactive tumors following anti-PD-1 treatment (19). Another study that used scRNA-seq revealed that following the use of ICIs combined with chemotherapy, the major pathological response (MPR) group presented more pronounced increases in Tem (CD8_GZMK), Trm (CD8_GZMB), and circulating effector T cells (CD8_STMN1) than the nonresponders (TN) (20). A previous meta-analysis evaluated the prognostic role of CD8+ tumor-infiltrating lymphocytes (TILs) among cancer patients receiving ICIs, including those with NSCLC. The results indicated that patients with high CD8+ T-cell infiltration had improved OS, PFS, and ORRs compared with those with lower levels of CD8+ T-cell infiltration. The quantity of CD8+ T cells in both the tumor and stroma was associated with OS and PFS in patients receiving ICIs treatment (21). In a retrospective study, the authors assessed the impact of baseline CD8+ TILs on patient prognosis. The results indicated that in the CRT + ICIs treatment group, a high density of CD8+ TILs (TIL high ≥ 100/mm²) was significantly associated with longer PFS (NR vs. 9.5 months; *p* = 0.002), whereas in the CRT alone group, CD8+ TILs had no effect on prognosis (22). In metastatic NSCLC patients receiving anti-PD-1 treatment, the response rate was only 16.7% when the number of CD8+ TILs was < 886/mm²; the response rate increased to 60% when the number of CD8+ TILs ranged from 886–1899/mm² (23). However, the thresholds for the level of CD8+ T-cell infiltration vary across different studies, which limits the application of CD8+ T cells in predicting the efficacy of ICIs. Additionally, not all CD8+ TILs can be reactivated by ICIs; some CD8+ T cells do not contribute to antitumor immunity (24, 25). In some studies, total CD8+ T cells could not predict the efficacy of ICI treatment. Furthermore, researchers have explored the ability of cells coexpressing CD8+ T cells and other cell surface markers to predict ICIs efficacy. A previous study revealed that CD39 can serve as a tumor-specific T-cell marker and that CD39+ CD8+ T cells can predict the response of NSCLC patients to PD-1 or PD-L1 blockade treatment, thus serving as an independent predictive indicator. The proportion of CD39+ CD8+ T cells was significantly higher in responders (partial response) than in nonresponders (stable disease or disease progression) (20). CD8+ T cells with high PD-1 expression reflect a high affinity for tumor antigens, indicating the important role of these cells in antitumor immunity. Compared with nonresponders, ICIs-treated responders have significantly higher levels of PD-1 expression on their CD8+ T cells (26). The finding that CD8+ T cells express PD-1 typically indicates that the cytotoxic function of these cells is suppressed. PD-1+ CD8+ TILs can continuously secrete CXCL13, thereby recruiting CXCR5-expressing B cells and Tfh cells into the TME, thus playing an important role in the formation of TLSs. The level of PD-1 expression in CD8+ T cells that exceeded that of healthy donor PBMCs was defined as PD-1T. In NSCLC patients, the proportion of PD-1T cells among responders to PD-1 blockade therapy was significantly greater than that among nonresponders, and PD-1 T cells were associated with improved OS (HR 0.16 (95% CI 0.05–0.52),*p* < 0.05) (27). Granzyme B (GZB) and Ki-67 expression in T cells represents T-cell activation and proliferation, respectively. Low expression of GZB and Ki-67 suggests that T cells are in a dormant state. PD-1 blockade can restore the effector function of these T cells. Among TILs, those with high CD3 expression and low GZB and Ki-67 expression benefit significantly from PD-1 blockade therapy (28).

#### 3.1.2 Immune-inhibitory cells

The tumor-suppressive microenvironment is influenced by various inhibitory immune cells, including myeloid-derived suppressor cells (MDSCs), tumor-associated macrophages (TAMs), regulatory T cells (Tregs), tumor-associated neutrophils (TANs), and cancer-associated fibroblasts (CAFs), along with inhibitory cytokines like IL-6, IL-8, and TGF-β. This suppressive environment may be linked to unfavorable outcomes in immunotherapy (16, 29). Immunosuppressive cells in peripheral blood (such as MDSCs) can predict the efficacy of ICIs therapy (29).Within the TME, inhibitory immune cells also contribute to forecasting the effectiveness of ICIs therapy. In one previous study, the authors used scRNA-seq to analyze the changes in T cells before and after PD-1 blockade treatment in NSCLC. They reported that the frequency of Tregs slightly reduced in responders, whereas the frequency of Tregs significantly increased in nonresponders. Furthermore, in Tregs, the expression levels of genes associated with immunosuppression, such as IL1R2, REL, and LAYN, are increased (19).ICIs therapy can reactivate CD8+ T cells by stimulating TCR and CD28 pathways and can similarly enhance the function of inhibitory immune cells like Tregs using the same mechanisms. Thus, the balance between these two cell types may impact the effectiveness of ICIs treatment. In NSCLC patients who respond to PD-1 blockade therapy, CD8+ T cells show high levels of PD-1 expression, whereas non-responders have Tregs with high PD-1 expression. The ratio of PD-1 high-expressing CD8+ T cells to PD-1 high-expressing Tregs serves as a predictor for the efficacy of ICIs therapy. Optimal results are observed when CD8+ T cells exhibit high PD-1 expression while Tregs display low PD-1 expression (26). Using scRNA-seq to analyze changes in macrophages before and after ICIs treatment, Macro_SPP1 macrophages were shown to promote tumor angiogenesis, In contrast, Macro_SELENOP is noted for its anti-inflammatory effects. Both subtypes of macrophages exhibit strong M2 (anti-inflammatory or tumor-promoting) characteristics and are classified as tumor-associated macrophages (TAMs). After ICIs treatment, the number of Macro_SPP1 macrophages decreased, whereas the number of Macro_SELENOP macrophages increased in NMPR patients. Senescent Neu_CCL3 neutrophils can recruit Macro_SPP1 macrophages, thereby forming an inhibitory TME. After ICIs treatment, the number of senescent neutrophils decreased in MPR patients, with the most severe depletion observed in senescent Neu_CCL3 cells, whereas the number of senescent neutrophils increased in NMPR patients. After receiving anti-PD1 treatment, CAF-related gene signatures were significantly more prevalent in patients with disease progression (PD) compared to those who achieved a complete response (CR) or partial response (PR) (30).

#### 3.1.3 Tumor-infiltrating B cells

Tumor-infiltrating T cells are often directly distributed within the tumor and make direct contact with tumor cells. In contrast, tumor-infiltrating B (TIB) cells are located primarily in TLSs (31).B cells located in TLSs are linked to the response of antitumor antibodies and the proliferation of CD4+ T cell clones. These B cells from TLSs boost the activity of effector T cells through cytokine production. In NSCLC patients with high-density TLS B cells, the proportion of activated and memory CD4+ T cells is greater, whereas the proportion of Tregs is lower. These findings suggest that TLS B cells are crucial for the prognosis of NSCLC patients and the antitumor T-cell response (32, 33). Researchers have reported that B cells and TLS-related genes (such as CXCL13) are associated with the efficacy of immunotherapy among patients with NSCLC receiving neoadjuvant immunochemotherapy (34). After neoadjuvant treatment with pembrolizumab combined with chemotherapy, tumors from major pathological response (MPR) patients presented a greater abundance of B cells than non-MPR patients did. Additionally, neoadjuvant immunotherapy can induce B-cell class switching and antibody responses (35).

Further research using sc-RNA seq was performed to analyze the TME of NSCLC patients after neoadjuvant anti-PD-1 therapy combined with chemotherapy. The authors reported that FCRL4+FCRL5+ B cells (atypical memory B cells) were located in TLSs, and the abundance of these B cells was significantly greater in MPR patients than in non-MPR patients. These B cells not only increase the efficacy of immunotherapy but also serve as predictive factors for the response of NSCLC patients to ICIs treatment (36). Additionally, studies on mouse models and human lung adenocarcinoma have shown that B cells present in TLSs can produce antibodies that target endogenous retroviruses (ERVs), thereby inhibiting tumor progression. ICIs treatment can expand the B-cell response to target ERVs, thereby increasing the antitumor capacity of ERV-reactive antibodies and prolonging survival in mouse models (37). Furthermore, previous research has indicated that effective immunotherapy relies on CXCL13-mediated TLS formation and that therapeutic administration of CXCL13 can be combined with ICIs to enhance antitumor immune responses (37).

### 3.2 Tertiary lymphoid structures

#### 3.2.1 Introduction to TLS

Secondary lymphoid organs (SLOs) are essential in triggering adaptive immune responses against tumors. During this process, dendritic cells with tumor antigens travel from the tumor site to the SLO. There, they present these antigens to T cells and B cells through the major histocompatibility complex (MHC), which activates effector T cells and memory B cells to inhibit tumor growth (38). Under the persistent influence of chronic inflammatory factors, TLSs with secondary lymphoid organ-like structures and functions may form in nonlymphoid tissues (39, 40). Guided by chemokines such as CXCL13 and cytokines such as IL-7, lymphocytes and myeloid cells gradually accumulate, thus forming TLSs that include components such as follicular dendritic cells, follicular helper T cells, B cells, T cells, mature dendritic cells, and fibroblastic reticular cells (40). TLSs can exist in the stroma (extratumoral), parenchyma (intratumoral), or infiltrative margins (peritumoral) of tumor tissues. They gradually develop from initial lymphocyte aggregation into mature TLSs. The formation of TLSs within tumor tissues has significant biological implications, as TLSs bypass the process of dendritic cells and lymphocytes migrating from the tumor site to secondary lymphoid organs (SLOs). This enables T cells and B cells to rapidly initiate immune responses near the tumor tissue, significantly shortening the time required for the immune response to activate (41).

TLSs encompass several components, such as adjacent zones for T cells and B cells, the presence of PNAd+ high endothelial venules (HEVs), B cell class switching, mature DC-Lamp+ dendritic cells located in the T-cell zone, and chemokine expression. However, it can sometimes be challenging to determine whether a TLS fully meets all of these criteria, and the structure of the TLS may vary depending on location and inflammatory stimuli. Therefore, some researchers have suggested that these structures with specific tissue characteristics can be collectively referred to as TLSs (42, 43). TLSs play a positive role in promoting antitumor immune responses. Generally, the presence of TLSs within tumor tissues is associated with better treatment responses and lower recurrence rates (40, 43). Given the potential impact of TLSs on the TME, researchers suggest that TLSs might be linked to the prognosis of patients undergoing tumor immunotherapy. Furthermore, inducing the formation of TLSs as an independent therapeutic approach or in combination with other (immune) therapies could enhance tumor treatment efficacy. Studies have shown that ICIs treatment can promote the development of TLSs within tumor tissues (44).

#### 3.2.2 Methods for evaluating TLSs

In-depth exploration of TLSs requires accurate assessment of TLSs. Hematoxylin and eosin (H&E) staining and immunohistochemical (IHC) staining are two commonly used methods for TLS evaluation. H&E staining of tumor tissue sections enables the morphological evaluation of the presence and localization of TLSs. In H&E-stained sections, TLSs appear as clusters of T cells surrounding clusters of B cells. When mature TLSs are present, germinal centers can be clearly observed, thus enabling the accurate identification of mature TLSs in H&E-stained sections. Although H&E staining provides an overview of tissue structure, it may not fully reveal the fine details of TLSs, and immature or early TLSs may be overlooked because of the lack of clear boundaries between B and T-cell regions, potentially leading to an underestimation of TLS quantity. In contrast, IHC staining can more accurately identify the presence and distribution of TLSs when specific antibodies are used to mark particular cell types and molecules. For example, B cells can be marked with CD20, T cells can be marked with CD3, and follicular dendritic cells (FDCs) can be marked with CD23 (**Table 1**). Methods such as H-DAB and H&E-DAB provide specific labeling of target antigens, thus offering practical grounds for a more accurate identification of TLS presence and maturity. IHC staining can accurately identify early TLSs on the basis of the aggregation of CD20 B cells and CD3 T cells. Furthermore, IHC staining can clearly reveal the formation of CD21+CD23+ FDC networks in secondary TLSs, as well as the follicular structure formed by CD20 B cells and CD23 FDCs, thus distinguishing these cells from early or primary TLSs. While IHC staining partially compensates for the shortcomings of H&E staining, traditional IHC typically enables the detection of only 1–2 markers on a single section. To enable the simultaneous observation of multiple markers on a single section, multiplex immunohistochemistry (mIHC) plays an important role in the assessment of TLSs (39, 40, 45, 46). Whole-slide imaging (WSI) technology combined with image analysis software provides the possibility for automated quantitative analysis of stained slices, thus enhancing the efficiency and accuracy of TLS assessment (47).

**Table 1:** Common cellular markers for frequently studied immune cells.

Common indicators used in TLS immunohistochemical staining	
B cells	CD20+ CD19+
GC B cells	AID Ki67
Naive B cells	TCL1A+
Memory B cells	CD27+ FCRL4+
Plasma cells	CD138 CD269
T cells	CD3 CD8 CD4
CD8-cytotoxic T-cell	GZMB
Dysfunctional CD8+ T cells	CXCL13+CD8+
Tfh cells	PD-1
Treg cells	FoXP3
Mature DCs	DC-LAMP
FDCs	CD21 CD23

Gene expression profiles can also be used for TLS assessment. By using RNA-seq to detect gene expression related to cell populations or chemokines, a comprehensive evaluation of TLS status can be achieved. In earlier studies, researchers reported that the expression profiles of 12 chemokines could predict the presence of ectopic lymphoid structures in invasive colorectal cancer and melanoma (48). In public databases, grouping these 12 chemokines to obtain TLS+ and TLS- cells can predict early recurrence of hepatocellular carcinoma (49). Other studies have also indicated that TLS-related gene expression profiles are positively correlated with the number of TLSs detected by IHC (50). Single-cell RNA sequencing (scRNA-seq) and spatial transcriptomics technologies can provide more in-depth transcriptomic data for TLS research. These methods can accurately assess TLSs and reveal the complex interactions and dynamic changes in TLSs within the tumor microenvironment, thus offering new perspectives for understanding the role of TLSs in tumor immunity (40).

**Figure 2:**
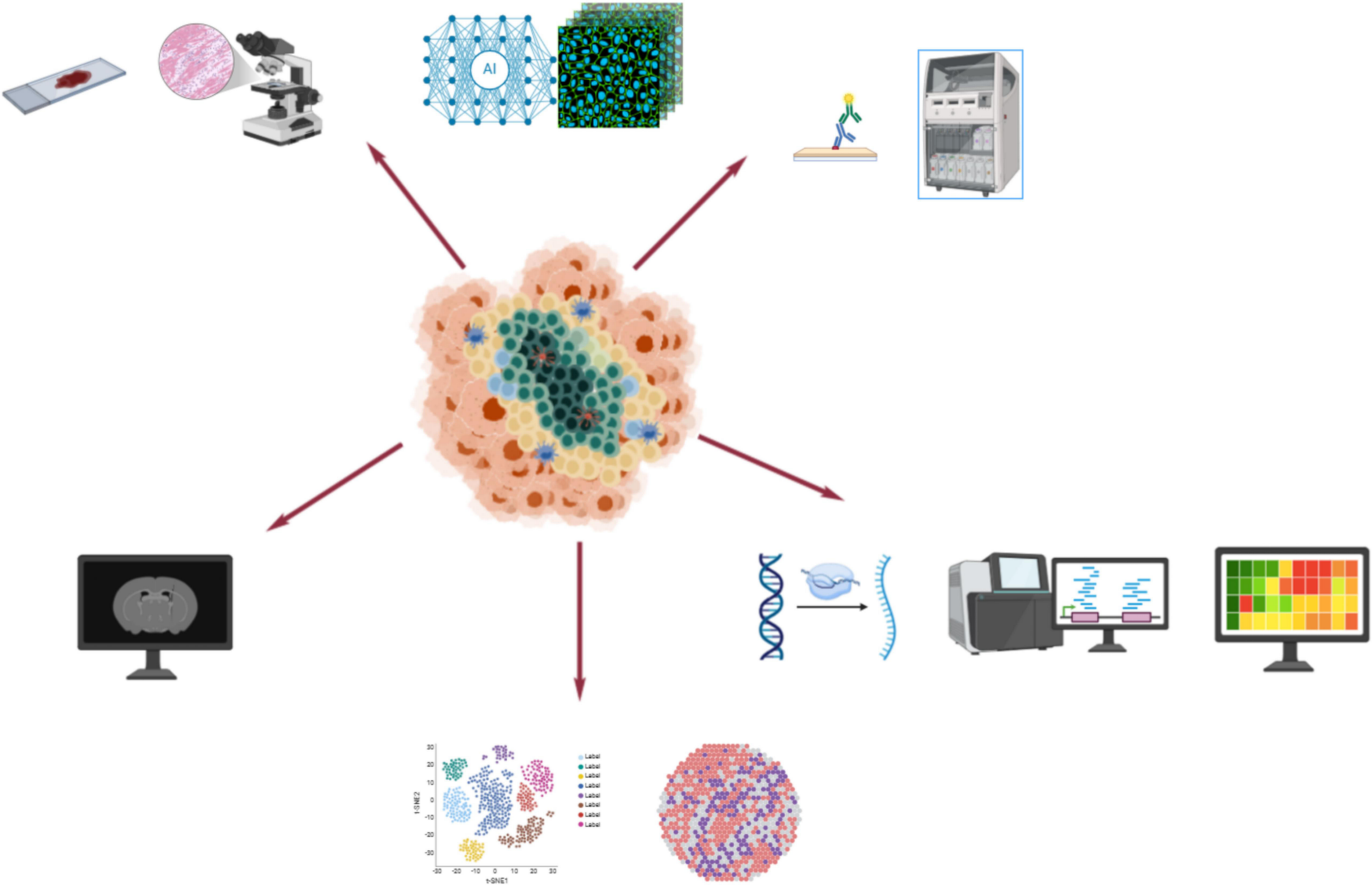
This figure presents a comprehensive overview of the diverse methodologies employed for the detection of TLS. The techniques highlighted include hematoxylin and eosin (H&E) staining for basic structural visualization, immunohistochemistry (IHC) staining for identifying specific cellular markers, bulk RNA sequencing for understanding gene expression profiles, single-cell sequencing for detailed cellular insights, spatial transcriptomics for mapping gene expression *in situ*, and radiomics analysis for non-invasive imaging assessments. These advanced techniques collectively enhance our ability to study and understand the complex role of TLS in various biological contexts.

Additionally, radiomics imaging methods, as novel approaches for assessing TLSs, are currently under active exploration. Combining imaging with advanced learning models and digital pathology can increase the visualization potential of TLSs. By analyzing images, TLS-related information can be obtained directly. However, this technology is still in the research stage (51) (**Figure 2**).

#### 3.2.3 TLSs’ capability to forecast the effectiveness of ICIs

TLSs are essential in the tumor immune microenvironment of NSCLC, as they create a vital local setting for activating immune cells and modulating immune responses. The presence of TLSs is closely associated with the outcomes for NSCLC patients. Tumors that are TLS-positive typically exhibit higher levels of tumor-infiltrating B cells, CD8+ T cells, and Th cells, indicating a more favorable immune environment (52). TLSs are closely related to the efficacy of ICIs treatment because they promote antitumor immune responses and enhance local immune responses.

**Figure 3:**
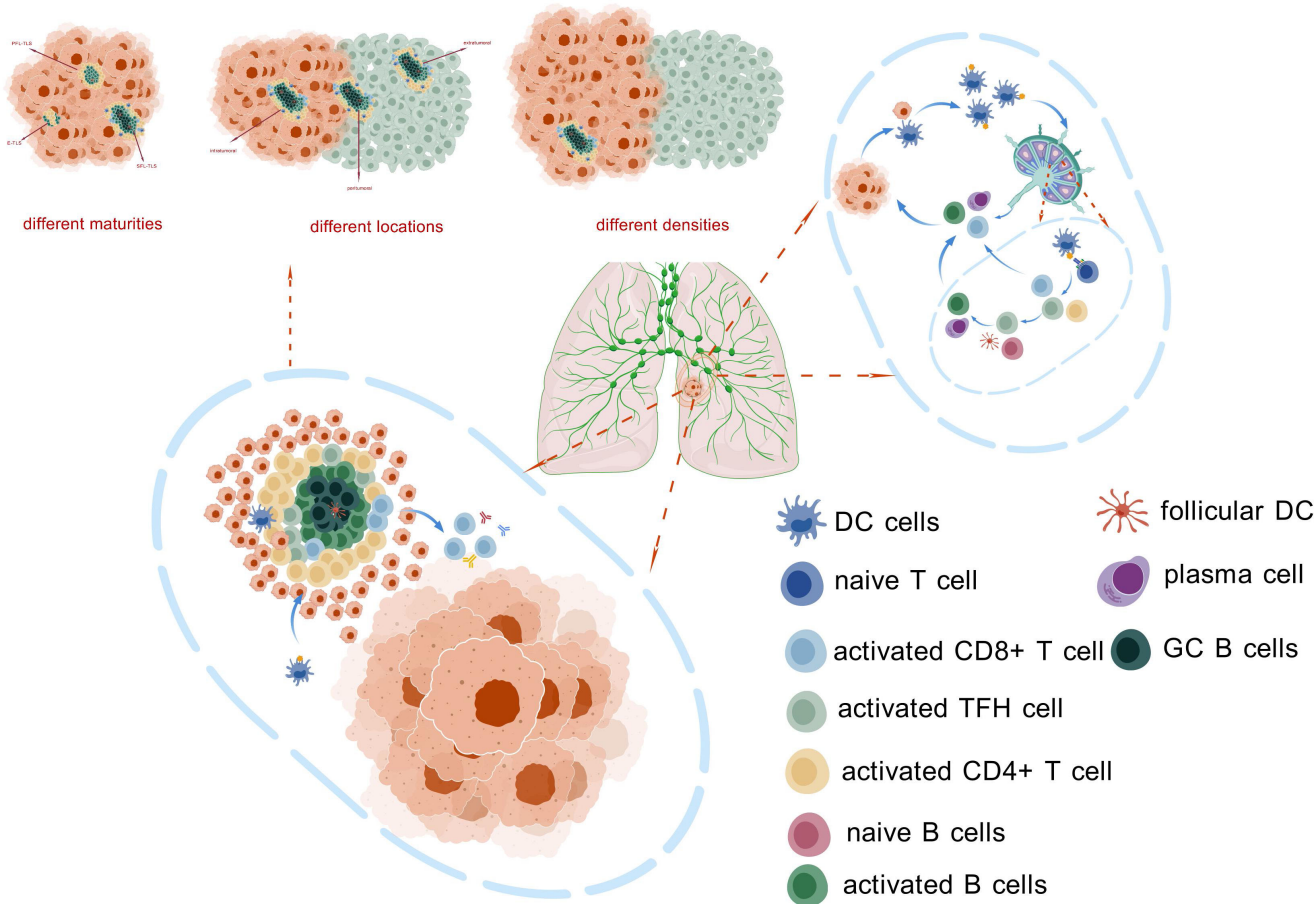
This figure illustrates the composition and function of TLS. These structures can be found at various locations within tumor tissues, exhibiting different levels of maturity and density. The presence of TLS facilitates the activation of antigen-presenting cells (APCs) and anti-tumor immune cells, enabling them to bypass the traditional migration process between tumor sites and draining lymph nodes. This allows for the rapid initiation of immune responses in close proximity to the tumor, potentially enhancing the effectiveness of anti-tumor immunity. The figure highlights the crucial role of TLS in modulating the tumor microenvironment and contributing to local immune regulation.

The maturity of TLSs reflects their functional effectiveness. Mature TLSs can effectively promote interactions between T cells and B cells, thereby triggering a stronger antitumor immune response. Additionally, immunotherapy can enhance the maturation of TLSs, thereby increasing tumor sensitivity to treatment. Therefore, assessing the maturity of TLSs may provide an important basis for optimizing immunotherapy regimens. The presence and density of TLSs are closely related to the patient’s immune response. Studies have shown that higher TLS density is often associated with better prognosis. The location of TLSs has been confirmed to be related to treatment efficacy and disease prognosis in various tumors; however, more in-depth research is needed to further elucidate the relationship between TLS location and NSCLC immunotherapy. Immunotherapy may improve treatment effectiveness by activating TLSs and enhancing local immune responses. This provides a potential biomarker for clinical practice, helping to identify patients who are likely to respond well to immunotherapy and improving treatment success rates (**Figure 3**, **Table 2**).

**Table 2:** The role of TLS with different maturation levels, locations, and densities in ICIs efficacy.

Level of maturity						
Treatment method	High-maturity group	Low-maturity group	Identification Method	Predictive Endpoint	Results	References
Neoadjuvant immunochemotherapy	PFL-TLS and SFL-TLS	Without TLS and E-TLS	H&E staining, immunofluorescence	MPR; DFS	In MPR patients, the number of mature TLSs was higher than in non-MPR patients, and the maturity of TLS can serve as an independent predictor of DFS.	(53)
Neoadjuvant immunochemotherapy	PFL-TLS and SFL-TLS	Without TLS and E-TLS	H&E staining	DFS	The DFS of the high maturity group is significantly longer than that of the low maturity group	(54)
Neoadjuvant immunochemotherapy	CD21+CD23+TLS	CD21+CD23-TLS	H&E staining multiplex immunohistochemistry	RFS; OS	The maturity of TLS can serve as an independent predictor of RFS.	(55)

Presence and density						
Treatment method	Group		Identification Method	Predictive Endpoint	Results	References
Immunotherapy	TLS-positive vs. TLS-negative		H&E staining	OS; PFS	Patients with TLS have greater benefits in terms of both OS and PFS.	(56)
Neoadjuvant immunotherapy	TLS-positive vs. TLS-negative		H&E staining, immunofluorescence	DFS	Compared to the TLS-negative group, the TLS-positive group demonstrated a significantly prolonged DFS.	(57)
Neoadjuvant immunotherapy	TLS-positive vs. TLS-negative		Immunostaining	EFS	The presence of TLSs is closely associated with long-term EFS	(58)
Neoadjuvant immunochemotherapy	High-density vs. low-density		Multiplex immunofluorescence	pCR; MPR	The density of TLSs is significantly higher in patients with pCR and MPR.	(59)
NEOADJUVANT immunochemotherapy	High-density vs. low-density		H&E staining, immunofluorescence	MPR; DFS	High abundance of TLSs is associated with better MPR and DFS.	(53)
neoadjuvant immunochemotherapy	High-density vs. low-density		H&E staining	DFS	Patients with ≥5 TLS have significantly prolonged DFS.	(54)
Neoadjuvant immunochemotherapy	High-density vs. low-density		Multiplex immunohistochemistry	MPR	Patients with MPR have a significantly higher density of TLSs.	(60)
Neoadjuvant immunotherapy	High-density vs. low-density		H&E staining	pCR; MPR	The number of TLSs is significantly higher in patients with pCR and MPR.	(61)
Neoadjuvant immunotherapy	High-density vs. low-density		NA	Depth of response	Patients with a better response to immunotherapy exhibit stronger TLS characteristics, and a higher TLS density is associated with better prognosis.	(62)

Location						
Treatment method	Group		Identification Method	Predictive Endpoint	Results	References
Neoadjuvant immunochemotherapy	Intratumoral 'vs. extratumoral		H&E staining multiplex immunohistochemistry	RFS; OS	The high density of intratumoral TLS is associated with a more favorable prognosis, while the density of extratumoral TLS does not show a significant correlation with clinical outcomes.	(55)

##### 3.2.3.1 The ability of TLSs with varying degrees of maturity to predict the efficacy of ICIs

In NSCLC treatment, the maturity of TLSs is strongly linked to the pathological response and effectiveness of neoadjuvant therapy. The maturation of TLSs starts with the development of HEVs and the clustering of T cells and B cells, which gradually develop into mature structures. This process is typically divided into three stages: early TLS (E-TLS), primary follicle-like TLS (PFL-TLS), and secondary follicle-like TLS (SFL-TLS). The E-TLS stage is characterized by the accumulation of T cells and B cells, but germinal centers and follicular dendritic cells have not yet formed. During the PFL-TLS stage, a network structure of CD21+ follicular dendritic cells (FDCs) is established within the B-cell region, although mature germinal centers have not yet appeared. The SFL-TLS stage marks the maturation of TLSs, featuring a complete CD21+CD23+ FDC network structure and active germinal centers, with a clear separation between the B-cell and T-cell zones. This structure is capable of supporting a comprehensive local immune response, including B-cell activation and antibody production (44, 63, 64).

In patients with lung adenocarcinoma, the presence of mature TLSs is closely associated with improved OS and DFS, and the presence of mature TLSs serves as an independent low-risk factor for lymph node (LN) metastasis (65). Research on lung squamous cell carcinoma has revealed that neoadjuvant chemotherapy significantly reduces the proportion of SFL-TLS and the size of germinal centers, suggesting that TLS maturation is suppressed. At this time, the predictive value of TLS density for survival in patients is lost (64).

Based on the maturity of TLSs, a previous study divided NSCLC patients into a low-maturity group (no TLSs and E-TLSs) and a high-maturity group (PFL-TLSs and SFL-TLSs). Among the 40 patients in the neoadjuvant immunochemotherapy group, 30 exhibited high-maturity TLSs; in the chemotherapy-only group of 41 patients, only 13 had high-maturity TLSs; and in the untreated control group of 40 patients, 25 displayed high-maturity TLSs. The data suggest that neoadjuvant chemotherapy may suppress the maturation process of TLSs, whereas ICIs treatment may induce TLS maturation (53). Further analysis revealed that in the neoadjuvant immunochemotherapy group, 45.0% (n=18) of patients achieved major pathological response (MPR), with 14 patients reaching pathologic complete response (pCR). In the neoadjuvant chemotherapy group, only 17.1% (n=7) of patients achieved MPR, with 2 patients reaching pCR. Patients with MPRs had higher levels of mature TLSs than non-MPR patients did, and TLS maturity was an independent predictor of disease-free survival (DFS) in the neoadjuvant immunochemotherapy group (53). The disease-free survival (DFS) of the high-maturity group (PFL-TLS and SFL-TLS) was significantly longer than that of the low-maturity group (no TLS and E-TLS), with median DFS durations of 34.07 months and 22.30 months, respectively (*p* = 0.024). These findings indicate that the maturity of TLSs is an important factor in predicting DFS after neoadjuvant therapy (54). Additionally, both the number of immature TLSs and mature TLSs was greater in the neoadjuvant immunochemotherapy cohort than in the neoadjuvant chemotherapy cohort. In the neoadjuvant immunochemotherapy group, the maturity of TLSs was identified as an independent predictor of recurrence-free survival (RFS), a finding that was not observed in the neoadjuvant chemotherapy group (55). After neoadjuvant immunotherapy, the presence of mature TLSs was associated with an increase in CD8+ T-cell density and enhanced infiltration of these cells into the tumor epithelial region, indicating an increase in immune activation within the tumor microenvironment (58). These observations suggest that, compared with the inhibitory effect of neoadjuvant chemotherapy on TLS maturation, neoadjuvant immunochemotherapy enhances TLS maturity. Furthermore, high-maturity TLSs are associated with an improved immune response in the tumor microenvironment and are correlated with better prognosis in patients receiving immunotherapy.

##### 3.2.3.2 The presence and density of TLSs as predictors of ICIs efficacy

In some tumor tissues, lymphocytes and myeloid cells aggregate and interact to form TLSs; however, TLSs are not present in all tumor samples, and their density can vary significantly. TLSs are significantly linked to tumor prognosis and are crucial in antitumor immune responses and the tumor microenvironment. A high TLS density often correlates with a stronger adaptive immune response within the tumor. Consequently, the presence and density of TLSs can predict the effectiveness of ICIs.

The IMpower110 trial was a clinical study that focused on patients with NSCLC and primarily evaluated the efficacy and safety of atezolizumab as a single-agent immunotherapy. The main endpoints of the trial included PFS and OS. In this study, TLSs were defined as lymphoid aggregates (LAs) containing one or more germinal centers. The results indicated that patients with TLSs experienced greater benefits in terms of both OS and PFS, suggesting that the presence of TLSs may be closely associated with the effectiveness of neoadjuvant immunotherapy (56). Additionally, TLS formation was more frequently observed in patients who responded to neoadjuvant immunochemotherapy, whereas it was rarely detected in nonresponders. Compared with the TLS-negative group, the TLS-positive group demonstrated significantly longer DFS, further emphasizing the importance of TLSs in neoadjuvant immunotherapy (57). Changes in the number and size of TLSs were observed after neoadjuvant immunotherapy, and the presence of TLSs was closely associated with long-term event-free survival (EFS) *(p* < 0.001) (58).

A greater density of TLSs was found to be associated with achieving pCR and MPR after neoadjuvant treatment among NSCLC patients who received neoadjuvant immunochemotherapy (66). In some studies, the authors explored TLSs in trials of neoadjuvant immunochemotherapy and neoadjuvant chemotherapy. Specifically, samples without TLSs were scored as 0; those with 1 to 2 TLSs were scored as 1; those with at least three TLSs were scored as 2; and samples with a high density of TLSs were scored as 3. The results indicated that the number of TLSs was significantly greater in the immunochemotherapy group than in the chemotherapy group. More importantly, a high number of TLSs was found to be associated with better MPR and longer DFS, further validating the potential value of TLSs in assessing the effectiveness of neoadjuvant treatment (53). Researchers have categorized samples into high-expression and low-expression groups on the basis of the number of TLSs observed in the field among NSCLC patients receiving neoadjuvant anti-PD-1/PD-L1 therapy combined with chemotherapy. Specifically, samples with fewer than 5 TLSs in 10 fields were defined as having low expression, whereas samples with ≥5 TLSs were defined as having high expression. The results revealed that patients with high expression of TLSs had significantly prolonged DFS (34.07 vs. 22.30 months, *p* = 0.041) (54). In studies of lung squamous cell carcinoma, patients who received neoadjuvant immunotherapy had a greater TLS density than did those who did not receive neoadjuvant treatment. In patients who respond more effectively to immunotherapy, TLS features are more evident, and a greater TLS density is linked to a more favorable prognosis (62).

Overall, the TLS density in the neoadjuvant immunochemotherapy cohort was generally greater than that in the neoadjuvant chemotherapy cohort. Neoadjuvant immunotherapy has the potential to promote TLS formation, suggesting that TLSs play an active role in enhancing patients’ immune responses. In NSCLC patients receiving neoadjuvant immunochemotherapy, those who achieved pCR and MPR were defined as the response group, whereas patients with poor treatment response were defined as the nonresponse group. The results indicated that after neoadjuvant treatment, TLS infiltration increased, and this increase was more pronounced in the response group than in the nonresponse group (59).

Combined analysis of inflammatory biomarkers and TLS results revealed that both TLSs and the PLR can be used to predict the MPR rate in NSCLC patients receiving neoadjuvant immunochemotherapy. Notably, using the combination of the PLR and TLS to assess the MPR in NSCLC patients is more accurate than using either indicator alone. These findings suggest that the combined use of TLSs and traditional biomarkers may increase the accuracy of prognostic assessment (67). In patients who achieved the MPR after neoadjuvant immunochemotherapy, the TLS density was significantly greater (60). In a study of patients with multiple primary lung cancers receiving neoadjuvant pembrolizumab treatment, researchers examined lesions at three sites. After neoadjuvant therapy, TLS formation was observed in all three types of nodules, and compared with nonresponsive nodules, responsive nodules exhibited a greater extent of TLS formation (68).

In a phase II clinical trial (NCT02259621) investigating neoadjuvant therapy, researchers reported that the number of TLSs was significantly greater in pCR and MPR patients than in NR patients (with ≥90% residual RVT in posttreatment samples), suggesting that TLS formation is positively correlated with a favorable treatment response (61). Furthermore, in patients receiving neoadjuvant durvalumab treatment, an analysis of surgical resection tumor samples posttreatment revealed a significant increase in TLS gene expression in MPR patients (69). After neoadjuvant treatment with pembrolizumab combined with chemotherapy, the gene expression of CXCL13 and 12 other chemokines was significantly elevated in tumor tissue. Additionally, mIHC observations revealed a marked increase in TLS density in tumor lesions following neoadjuvant immunotherapy (35). In summary, TLSs play a crucial role in the treatment of NSCLC with ICIs. Their density is significantly associated with pathological responses, such as pCR and MPR, and a high TLS density typically indicates a better prognosis. These findings suggest that TLSs are not only effective biomarkers for assessing immune therapy responses but also potential predictors of long-term survival in patients.

##### 3.2.3.3 The spatial location of TLSs predicts the efficacy of ICIs

TLSs are highly heterogeneously distributed in NSCLC, as they can form in multiple regions of the tumor tissue, including the stroma (extratumoral), parenchyma (intratumoral), and invasive margins (peritumoral) (41). This variation in spatial distribution not only indicates the tumor’s biological behavior but may also significantly impact the treatment response.

In studies of NSCLC, the positivity rate of TLSs within the tumor is usually significantly higher than that in the peritumoral and extratumoral regions, and the average TLS density within the tumor is also markedly greater than that in other areas. The location of TLSs within tumor tissue is not only related to tumor growth and metastatic potential but also closely associated with the clinical and pathological features of patients. For example, a high density of TLSs in the peritumoral or extratumoral regions may be linked to tumor invasiveness and unfavorable prognostic factors. In other types of cancer, such as liver cancer and cholangiocarcinoma, the different locations of TLSs also have been shown to exert various impacts on tumor growth and treatment response (70). The localization of TLSs suggests that they play complex roles in the tumor microenvironment. In patients receiving neoadjuvant immunochemotherapy for NSCLC, a high density of intratumoral TLSs was associated with a more favorable prognosis, whereas the density of extratumoral TLSs is not significantly correlated with clinical outcomes in the two treatment cohorts (55). In the context of immunotherapy, the localization of TLSs may have significant implications for the selection of treatment strategies and the prediction of treatment efficacy. For example, immunomodulatory therapies targeting intratumoral TLSs may more effectively enhance local immune responses, thereby improving the efficacy of neoadjuvant treatment. The localization of TLSs could help identify patients who are most likely to benefit from specific therapies. However, research on the localization of TLSs in NSCLC is still relatively scarce, thus highlighting the urgent need for more systematic research to further elucidate their specific roles in tumor progression and treatment response.

## 4 Tumor genomic alterations

The tumor cell genome can undergo changes, and during DNA replication, a DNA damage repair (DDR) system exists that identifies and corrects replication errors. Defects in the DDR system can lead to the continuous accumulation of genomic abnormalities, which is a significant cause of tumor development (71, 72). Among them, mismatch repair deficiency (dMMR) can lead to microsatellite instability (MSI) (5, 73); TMB quantifies somatic mutations in tumor cells. Typically, TMB counts nonsynonymous single-nucleotide mutations, but in some cases, TMB may also include synonymous mutations as well as insertions and deletions (indels) (73, 74). All of the above genomic changes can generate neoantigens, and peptide segments containing these neoantigens can be presented on the cell surface, where they may be recognized by T cells (74). The recognition of neoantigens generated by mutations in T cells is an important reason for the efficacy of anti-PD-1 therapy (75). Therefore, tumor genomic alterations and neoantigens are hypothesized to be predictive markers for the efficacy of ICIs among NSCLC patients. In NSCLC, mismatch repair deficiency and high microsatellite instability are relatively rare, but it is one of the tumors with the highest TMB (74). The somatic mutation differences between smokers and nonsmokers are significant, resulting in a wide range of TMBs in NSCLC (75, 76). Multiple studies have confirmed that TMB serves as a predictive marker for ICIs treatment efficacy in NSCLC patients (**Table 3**), independent of PD-L1 expression levels (81).

**Table 3:** The predictive role of TMB in ICIs efficacy.

Immunotherapy approaches	TMB cutoff	Results	References
Pembrolizumab	Experimental group: Median nonsynonymous mutation burden: 209 Validation group: Median nonsynonymous mutation burden: 200	Experimental group: DCB: 73% vs. 13% ORR: 63% vs. 0% PFS: 14.5 vs. 3.7 months Validation group: DCB: 83% vs. 22%	(75)
Nivolumab combined with ipilimumab	≥10 mutations per megabase	1-year PFS: 42.6% vs. 13.2% (chemotherapy) Median PFS: 7.2 vs. 5.5 months ORR: 45.3% vs. 26.9%	(77)
Nivolumab combined with ipilimumab	Median: 158 mutations	ORR: 51% vs. 13% DCB: 65% vs. 34%	(78)
Anti-PD-1/PD-L1	High TMB: ≥20 mutations/Mb Medium/Low TMB: <20 mutations/Mb	OS: 16.8 vs 8.5 months Duration of treatment: 7.8 vs. 3.3 months	(79)
PD-1/PD-L1 inhibitors combined with CTLA-4 inhibitors	TMB threshold: 16	High TMB individuals Prolonged PFS (HR = 0.54, 95% CI: 0.46–0.63, *p* < 0.001) Prolonged OS (HR = 0.70, 95% CI: 0.57–0.87, *p* = 0.001) Higher ORR (OR = 3.14, 95% CI: 2.28–4.34, *p* < 0.001)	(80)
Nivolumab	Low TMB: 0-100 Medium TMB: 100-242 High TMB: ≥243	Response rate: 47% vs. 28% (chemotherapy) PFS: 9.7 vs. 5.8 months	(81)
Pembrolizumab	TMB threshold: 175 mutations/exome	High TMB group KEYNOTE-010: Median OS: 14.1 vs. 7.6 months (chemotherapy) Median PFS: 4.2 vs. 2.4 months ORR: 23.5 vs. 9.8 KEYNOTE-042: Median OS: 21.9 vs. 11.6 months	(82)
Atezolizumab	1L: Median TMB ≥ 9 mutations/MB, High TMB ≥ 13.5 mutations/MB 2L+: Median TMB ≥ 9.9 mutations/MB, High TMB ≥ 17.1 mutations/MB	1 L: ≥9/MB vs.≥13.5/MB OS:0.79 vs. 0.45 PFS:0.58 vs. 0.54 2 L+:≥9.9/MB vs. ≥17.1/MB OS: 0.87 vs. 0.7 PFS: 0.64 vs. 0.5	(83)
Duvelisib Duvelisib combined with Tislelizumab	tTMB:10 mut/Mb	Median OS in the tTMB ≥ 10 mut/Mb group: Duvelisib vs. Duvelisib combined with Tislelizumab vs. chemotherapy 16.6 months vs. 18.6 months vs. 11.9 months”	(84)

Detection of the average copy number variation (CNVA) of chromosome fragments reflects genomic instability by measuring the average copy number changes in small chromosome segments. In studies of NSCLC patients receiving immunotherapy, the high-CNVA group presented elevated PD-L1, CD39, and CD19 expression levels, as well as increased infiltration levels of CD8+ T cells and CD3+ T cells. These findings indicate a high immune infiltration status in the tumor tissue and suggest that the CNVA may serve as a potential alternative indicator for predicting the efficacy of immunotherapy in patients with NSCLC (85).

Additionally, specific gene mutations in NSCLC influence the effectiveness of ICIs. Different gene mutations correspond to varying levels of tumor PD-L1 expression and TMB. For instance, EGFR-mutant NSCLC generally has lower levels of both PD-L1 expression and TMB, while KRAS-mutant NSCLC often shows higher levels. EGFR mutations are common in NSCLC, with the most prevalent forms being exon 19 in-frame deletions and the L858R point mutation in exon 21, accounting for over 90% of all EGFR mutations. EGFR mutations can influence the components of the TME. For example, they can upregulate chemokines such as CXCL10 and CCL2 through the PI3K-AKT-mTOR pathway, further recruiting Tregs and M2 macrophages while reducing the infiltration of CD8+ T cells, Th cells, NK cells, and M1 macrophages. This process promotes the formation of an immunosuppressive microenvironment, which is detrimental to the efficacy of ICIs (86, 87). In NSCLC subtypes with changes in EGFR, HER2, ALK, ROS1, or RET, solely targeting the PD-L1–PD-1 axis provides limited benefits. In contrast, those with KRAS and TP53 co-mutations tend to have better responses to PD-L1-PD-1 blockade, whereas patients with KRAS mutations alongside STK11 and/or KEAP1 often experience poorer outcomes with ICIs treatment (86).

Epigenetic modifications can influence gene expression without altering the DNA sequence itself. These modifications not only play a crucial role in the development and progression of tumors but also have the potential to predict the efficacy of ICIs to some extent. Through mechanisms such as DNA methylation and histone modifications, epigenetic processes can impact the expression of genes associated with immune checkpoints and tumor-associated antigens, thereby affecting the efficacy of ICIs (16, 88).For instance, a study shows that genome-wide methylation patterns characterized by promoter hypermethylation can predict the efficacy of immunotherapy response in NSCLC (89). In another study involving stage IV NSCLC patients treated with anti-PD-1 agents, a DNA methylation signature known as EPIMMUNE was found to correlate with both PFS and OS (90). Histone deacetylases (HDACs) are a group of enzymes that remove acetyl groups from histones, playing a significant role in regulating gene expression, which is crucial for cellular processes such as proliferation, differentiation, and apoptosis. HDAC8 (histone deacetylase 8) has been shown to restore the effector functions of CD8+ T cells, thereby enhancing the therapeutic effects of anti-PD-1 treatment in NSCLC (91). Additionally, research has indicated that the expression of HDAC6 can serve as a prognostic marker for NSCLC patients undergoing ICIs treatment. Furthermore, the combination of HDAC inhibitors with PD-1 inhibitors has demonstrated the potential to reduce tumor growth rates and create a more favorable TME for cytotoxic T lymphocytes (92). Moreover, the integration of epigenetic alterations with liquid biopsy technologies presents new avenues for predicting the efficacy of ICIs in NSCLC. Biomarkers based on DNA methylation in ctDNA show great promise for applications in screening, early diagnosis, and predicting as well as monitoring responses to specific therapies (93). While existing studies have highlighted the relationship between ctDNA methylation and targeted therapies, the predictive role of ctDNA methylation regarding ICIs efficacy warrants further investigation (93). In summary, epigenetic modifications are closely related to anti-tumor immune responses, and ongoing research in this field may lead to the discovery of novel biomarkers for predicting the efficacy of ICIs.

## 5 Transcriptome signatures

Biomarkers based on gene expression are extensively utilized in oncology. The use of transcriptomic signals to reflect the expression levels of specific genes can predict the efficacy of ICIs treatment. This can be achieved through a comprehensive analysis of transcriptional signals related to T-cell activation, antigen presentation, and the IFNγ pathway. IFNγ is a key regulator of the immune system and plays a crucial role in antitumor immunity. It is also closely linked to PD-L1 expression levels. Research on different tumors has demonstrated that mRNA levels related to IFNγ can predict the effectiveness of PD-1 blockade therapy (94). In a group of NSCLC patients undergoing treatment with durvalumab, those with increased expression levels of genes encoding IFNγ, CD274, LAG3, and CXCL9 were defined as IFN-γ+. Compared with IFN-γ− patients, IFN-γ+ patients exhibited significant improvements in the ORR, median OS, and median PFS (95)., Mutations in genes associated with the IFNγ signaling pathway can lead to poor efficacy of ICIs treatment (5). In NSCLC patients receiving atezolizumab treatment, those with higher levels of T-cell effector and IFNγ-related gene expression experienced prolonged overall survival (96). The IFNG gene encodes IFN-γ, and NSCLC patients treatment with receiving nivolumab treatment who had a high expression of IFNG demonstrated significantly prolonged PFS (97). The GDPLichi score, which is composed of seven DNA damage repair-related genes (DUT, MGMT, POLH, RAD1, RAD17, TYMS, and YWHAG), categorizes patients into high-risk and low-risk groups. Compared with the low-risk group, the high-risk group presented significantly greater TMB, higher neoantigen levels, and higher expression levels of PD-L1, PDCD1, and CTLA4. Therefore, patients in the high-risk group are more sensitive to immunotherapy, and the GDPLichi model can be used to predict the efficacy of ICIs in patients with lung adenocarcinoma (72). In NSCLC patients undergoing anti-PD-L1 therapy, CSF1R and HCST expression levels showed a positive correlation with PD-L1 levels and a high presence of CD8+ T cells, indicating their potential predictive value for prognosis (98). Additionally, TCR coexpressed gene signatures have also been confirmed as predictive factors for ICIs treatment in NSCLC, with elevated expression levels indicating a better prognosis (99).

## 6 Circulating biomarkers

**Figure 4:**
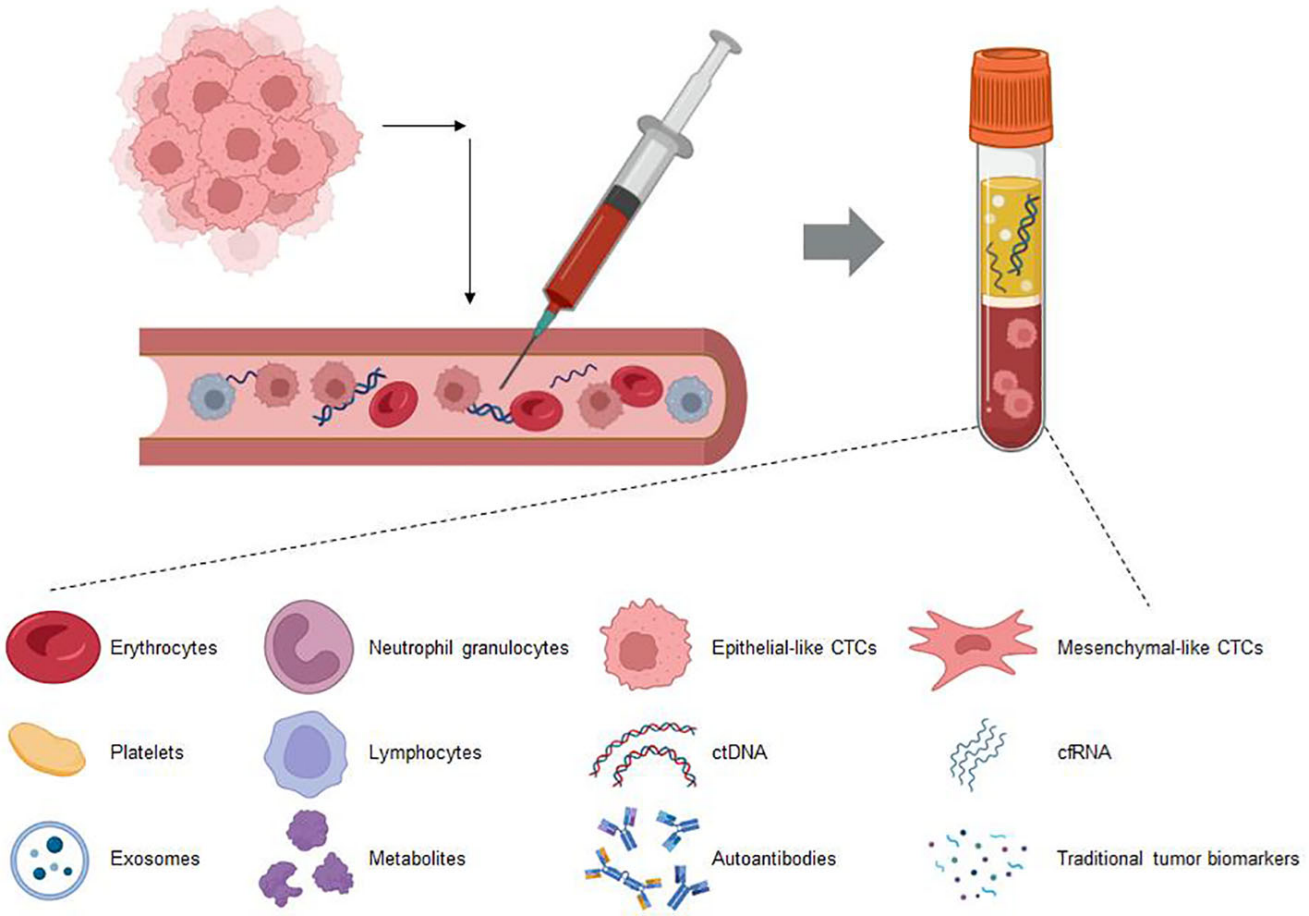
This figure showcases the various biomarkers detectable in blood circulation through liquid biopsy techniques. These biomarkers include circulating tumor DNA (ctDNA), exosomes, circulating immune cells, circulating tumor cells (CTCs), and soluble proteins. Liquid biopsy provides a minimally invasive method to capture these diverse components, offering valuable insights into the molecular and cellular dynamics within the body. This approach holds significant promise for early diagnosis, monitoring disease progression, and tailoring personalized treatment strategies in oncology.

Circulating biomarkers refer to biomolecules that are present in the blood or other bodily fluids, such as ctDNA and exosomes, that can reflect tumor characteristics or treatment responses (**Figure 4**). They provide a noninvasive method for early diagnosis, prognosis, and monitoring treatment efficacy, thus representing a significant advancement in clinical practice (100).Circulating tumor DNA (ctDNA) is composed of fragments shed into the bloodstream by tumor cells. Analyzing ctDNA can reveal tumor-specific genetic changes, and tracking ctDNA levels or modifications (like ctDNA methylation) can indicate the effectiveness of ICIs therapy in NSCLC. The blood tumor mutational burden (bTMB) is assessed through ctDNA analysis. In the MYSTIC trial, NSCLC patients with bTMB ≥ 20 mut/Mb who received combination immunotherapy showed enhanced overall survival (84). Previous research indicated that a high ctDNA mutational burden was associated with better efficacy of ICIs treatment across various cancer types, including NSCLC (101). Moreover, ctDNA levels can also serve as a predictive biomarker. In the B-F1RST trial, NSCLC patients receiving atezolizumab treatment with low levels of ctDNA (max somatic allele fraction (MSAF) <1%) had ctDNA amounts that were insufficient for accurate bTMB assessment. However, compared with patients with an MSAF ≥1%, the ORR was significantly better among those with an MSAF <1% (34.5% vs. 10.1%) (102). In a study on ICIs treatment for metastatic NSCLC, the authors quantified ctDNA by measuring the allele fraction of cancer-associated somatic mutations in plasma, defining a ctDNA response as a reduction of more than 50% in the mutant allele fraction. The results indicated that the ctDNA response was associated with improved PFS and OS, and it could predict treatment efficacy at an earlier stage (103). NSCLC patients with molecular residual disease (MRD) following chemoradiotherapy received consolidation ICIs treatment. During the treatment period, two different ctDNA response types were observed: an increase in ctDNA or a decrease in ctDNA. Patients with elevated ctDNA concentrations showed poor response to ICIs treatment, with all of them experiencing disease progression within 4.5 months after the initiation of consolidation therapy. Conversely, patients with decreased ctDNA concentrations benefited from consolidation ICIs treatment (104). In NSCLC patients undergoing immunochemotherapy, alterations in the ctDNA allele fraction (AF) correlated with imaging changes and long-term clinical outcomes. Patients with a reduction in AF had a significantly higher response rate compared to those with an increased AF (60.7% vs. 5.8%, *p* = 0.0003). Moreover, both median PFS and OS were improved (105). The abovementioned study demonstrated the value of ctDNA for predicting the efficacy of ICIs treatment in NSCLC patients. However, currently, there is no standard process for the collection and handling of ctDNA, and there are limited clinical application data, which restricts its use in efficacy prediction (106).

Circulating immune cells also have a certain predictive function for the efficacy of ICIs treatment. For example, in NSCLC patients treated with ICIs, the number of CD4+CD25+CD127loFoxP3+ Treg cells significantly decreased compared with that at baseline in patients with pseudoprogression one week after treatment, whereas the number of these cells markedly increased in patients with hyperprogression. In the responder group, both CD4+CD25+CD127loFoxP3+ Treg cells and PD-1+CD4+CD25+CD127loFoxP3+ Treg cells were significantly reduced (107).In NSCLC patients treated with anti-PD-1 antibodies, elevated baseline levels of circulating CD4+CCR9+, CD4+CCR10+, or CD8+CXCR4+ T cells were linked to poorer overall survival (15.7 vs. 35.9 months, HR 0.16, *p* = 0.003; 22.0 vs. not reached, HR 0.10, *p* = 0.003; and 22.0 vs. not reached, HR 0.29, *p* = 0.02) (108). In a cohort of advanced NSCLC patients treated with atezolizumab, an increase in the lymphocyte ratio was observed in patients with disease control, whereas a significant decrease was noted in patients with disease progression. Additionally, a decrease in circulating CD4+ and CD8+ T cells and an increase in Tregs and MDSCs were observed in the disease progression group, whereas the opposite changes in circulating immune cells were observed in the disease control group (109)In NSCLC patients undergoing nivolumab treatment, an elevated central memory/effector T-cell ratio was linked to better PFS, indicating higher tumor PD-L1 expression levels. Conversely, an increase in exhausted cells and a reduction in memory effector CD8+ T cells were associated with disease progression (110, 111). The neutrophil-to-lymphocyte ratio (NLR) is determined by comparing the absolute numbers of circulating neutrophils to lymphocytes. The NLR is closely related to the innate immune system and can reflect the tumor status (112). In NSCLC patients receiving neoadjuvant ICIs therapy, a decrease in the NLR of more than 10% after four weeks of treatment has been shown to be associated with tumor regression and the MPR. Additionally, patients with a decreased NLR had improved PFS and OS (113–115). In NSCLC patients with a PD-L1 TPS ≥50% and without EGFR or ALK mutations treated with pembrolizumab, those with a dNLR <2.6 had a significantly improved ORR, median PFS, and median OS compared with those with a dNLR ≥2.6 (116). During the first six weeks of nivolumab treatment, early increases in cfDNA and the NLR indicate poorer survival outcomes in advanced NSCLC patients, suggesting the potential role of these biomarkers in real-time monitoring of immunotherapy resistance (117).Moreover, a higher myeloid-to-lymphoid cell ratio (M:L) and elevated absolute neutrophil count were linked to shorter PFS and OS (118). A low platelet-to-lymphocyte ratio (PLR) and a low monocyte-to-lymphocyte ratio (MLR) are associated with better PFS (119).Furthermore, there are additional potential predictive biomarkers in circulation. Blood-based soluble immune checkpoint-related proteins, like soluble PD-L1 (sPD-L1), are linked to the prognosis of advanced lung cancer and might serve as potential indicators for predicting the effectiveness of ICIs (120).The potential of sPD-L1 as an alternative marker for PD-L1 TPS is still being studied. After treatment with nivolumab, either an increase or stabilization in plasma sPD-L1 levels has been associated with a more favorable prognosis (121). Increased preoperative sPD-L1 levels were found to be associated with poor prognosis (122). Serum granzyme B is released by cytotoxic CD8+ T cells and NK cells. An analysis of stage IV NSCLC patients receiving nivolumab treatment revealed that patients with lower baseline serum granzyme B levels had poorer PFS and OS than those with higher levels (123). Circulating tumor cells (CTCs) refer to cancer cells released from the primary tumor that enter the bloodstream or lymphatic system and may form metastases in other parts of the body. An analysis of CTCs in advanced NSCLC patients receiving nivolumab treatment revealed that the presence of CTCs or the expression of PD-L1 on the surface of CTCs could reflect treatment efficacy: patients with PD-L1+ CTCs were more likely to experience disease progression. However, owing to the small sample size of the study, further experiments are needed for validation (124).Extracellular vesicles (EVs), such as exosomes and microvesicles, carry bioactive molecules and are crucial in cellular communication (125). Previous research has indicated that EVs derived from tumor tissue can serve as noninvasive biomarkers. Higher levels of costimulatory molecules such as CD9, CD81, and CD63 in EVs are associated with an ICIs response and a better ORR (126). EVs can carry small molecular substances such as miRNAs, which have also been confirmed to be associated with ICIs efficacy.EV-miR-625-5p has been recognized as an independent biomarker for predicting the response to ICIs in NSCLC patients with PD-L1 expression levels of 50% or higher (127, 128).EVs offer a novel method for detecting PD-L1, where higher levels of exosomal PD-L1 are linked to improved ORR and OS (129).Research on patients with malignant melanoma and NSCLC undergoing anti-PD-1 antibody therapy showed that two months post-treatment, the PD-L1 mRNA levels in plasma exosomes significantly decreased in responders. In contrast, there was no notable change in patients with stable disease, while those with disease progression experienced a significant increase (130). Researchers have proposed a biosensor for the quantitative detection of the EVs PD-1/PD-L1 mRNA (Au SERP); this method achieved an accuracy of 72.2% in distinguishing between ICIs responders and nonresponders (131). EVs have broad application prospects in the exploration of noninvasive biomarkers.

## 7 Microbiome

The microorganisms that reside in the human gut, skin, and other mucosal surfaces are collectively referred to as the microbiome. They engage with the human immune system and contribute to the establishment and progression of both innate and adaptive immunity (132). Recent studies have uncovered a connection between the microbiome and the effectiveness of ICIs therapy. Research on the microbiome in lung cancer has focused primarily on gut microorganisms. Currently, studies have also revealed the impact of lower respiratory tract microbes on lung cancer and their potential ability to predict lung cancer treatment outcomes (133–135). Pulmonary microorganisms in the human body are associated with various diseases, and the composition of the lung microbiome differs across lung cancer patients. Research has shown that *Veillonella dispar* predominates in lung cancer patients with high PD-L1 expression, whereas the abundance of *Neisseria* species is significantly greater in patients with low PD-L1 expression. Furthermore, *V. dispar* is prevalent among those who respond to immunotherapy, while *Haemophilus* influenzae and *Neisseria perflava* are more frequently found in non-responders (136). The microbiome is linked to immune-related adverse events (iRAEs) in lung cancer patients receiving immunotherapy. Those who did not encounter iRAEs had a more diverse gut microbiota, including *Bifidobacterium* and *Desulfovibrio*. In patients who respond to chemotherapy combined with immunotherapy, there is an increase in *Clostridiales* and a decrease in *Rikenellaceae (* 137). In NSCLC patients receiving ICIs treatment, the abundance of *Akkermansiaceae* is greater in those with stable disease and partial response than in those with disease progression (138, 139). In patients receiving ICIs treatment who never use antibiotics, the α diversity of the gut microbiome is associated with OS. In patients with a good ORR and PFS greater than 6 months, *Ruminococcaceae UCG 13* and *Agathobacter* were more abundant. Additionally, there are differences in the gut microbiota between patients with high-grade and low-grade iRAEs (140). In NSCLC patients receiving anti-PD-1 treatment, those with higher microbial diversity have prolonged PFS compared with those with low microbial diversity. There are also differences in the gut microbiota composition between responders and nonresponders (7). However, the microbiome exhibits significant interindividual variability and is easily influenced by environmental factors, which limits its application in predicting the efficacy of ICIs.Advancements in detection technologies are necessary to assess the potential of the microbiome in personalized treatment.

## 8 Discussion

NSCLC, the most common form of lung cancer, significantly impacts global health. Immunotherapy is becoming an increasingly vital part of NSCLC treatment. However, a majority of patients still fail to respond favorably to immunotherapy. As a result, ongoing research is largely concentrated on discovering biomarkers that can forecast the effectiveness of immunotherapy. This helps in selecting patients who are more likely to benefit, thereby improving the overall success rate.

The TME is the local environment in which tumor cells are directly embedded, and its components and functions play crucial roles at various stages of tumorigenesis. Immunotherapy can cause localized alterations in the TME, and the components of this microenvironment may also play important roles in predicting the efficacy of immunotherapy. Tumor-infiltrating lymphocytes are immune cells that infiltrate tumor tissues, and the interactions between these immune cells can regulate the immune state of the tumor microenvironment, which is closely correlated with patient prognosis. As the main cell type involved in antitumor immunity, high infiltration of CD8+ T cells is associated with better outcomes in immunotherapy. Additionally, the expression of other surface markers on CD8+ T cells may reflect changes in their function, and certain types of CD8+ T cells (such as CD39+CD8+ T cells and PD-1+CD8+ T cells) may predict benefits from immunotherapy more effectively than total CD8+ T-cell infiltration alone. There are also immunosuppressive cells in the TME, such as Tregs, TANs, TAMs, and CAFs. Typically, CAFs are considered to have multiple tumor-promoting functions, including promoting tumor cell proliferation and angiogenesis in tumor tissues, and are associated with tumor invasion, metastasis, and drug resistance (141). However, recent results from scRNA-seq and spatial transcriptomics analyses have shown that certain subtypes of CAFs are closely associated with the formation of TLSs, which can recruit B cells and T cells, thus playing a significant role in antitumor immunity (142, 143). Owing to the significant heterogeneity of CAFs, more in-depth studies of CAFs based on single-cell and spatial transcriptomics analyses are needed. Tumor-infiltrating B cells are found mainly in TLSs, where they can produce antitumor antibodies and enhance effector T-cell responses. Higher B-cell infiltration correlates with better prognosis. Moreover, studies have shown that in patients with advanced NSCLC who have liver metastases undergoing immunotherapy with ICIs, the organ-specific response rate (OSRR) and organ-specific disease control rate (OSDCR) of liver metastases are lower than those of lung tumors. Further research has found that compared to lung tumors, the infiltration ratios of CD8+ T cells and CD56dim+ NK cells in liver metastases are significantly reduced (*p* = 0.036 and *p* = 0.016). Additionally, in liver metastases, the expression of immune activation-related genes (such as CD8A, LCK, and ICOS) is downregulated, and liver metastases exhibit more pronounced immunosuppressive characteristics, such as a higher proportion of Tregs and a lower capacity for effector T cell recruitment. These findings further emphasize the important role of the TME components in the efficacy of ICIs. The immunosuppressive microenvironment of liver metastases may limit the effectiveness of ICIs therapy. For example, the lower infiltration of CD8+ T cells and NK cells in liver metastases, along with the downregulation of immune activation-related genes, may hinder the effective activation of anti-tumor immune responses through immunotherapy. Given the immunosuppressive features of liver metastases, the research suggests that combination therapies (such as ICIs with anti-angiogenic agents or adoptive cell therapy) may be more beneficial in improving the effects of immunotherapy. Immune treatments targeting liver metastases need to consider their unique immunological microenvironment characteristics and develop more precise therapeutic strategies (144). TLSs are aggregates of immune cells that form within tumor tissues and exhibit structures and functions similar to those of secondary lymphoid organs. The formation of these structures indicates a more favorable immune state in the tumor, allowing immune cells to quickly initiate immune responses near the tumor and play a significant role in antitumor immunity. Their maturation status, presence, density, and location can partially predict the efficacy of immunotherapy. Compared with immature TLSs, mature TLSs have active germinal centers that support comprehensive local immune responses, and the presence of mature TLSs in tumor tissues can better predict the efficacy of immunotherapy. Not all tumor tissues can develop TLSs, and a high density of these structures suggests a better response to immunotherapy. TLSs can be found within the tumor, in the surrounding tissue, or outside the tumor; this spatial distribution reflects the biological behavior of the tumor and may also influence the treatment response. A high density of TLSs within the tumor is associated with better outcomes in immunotherapy. As an immune checkpoint molecule, PD-L1 binds to its ligand PD-1 to inhibit T-cell activity, and the expression level of PD-L1 is currently the most widely accepted and utilized biomarker for predicting the efficacy of ICIs. When PD-L1 is highly expressed, patients tend to have better outcomes following immunotherapy. Although the TPS is widely used to predict ICIs response, PD-L1 detection is typically based on IHC, which relies on the quality of tumor samples. Additionally, different laboratories use varying staining standards, and establishing a cutoff for PD-L1 is also a challenge. Therefore, the use of PD-L1 alone as a predictive indicator may not yield satisfactory results (145, 146). In squamous cell carcinoma, IHC detection of PD-L1 levels does not effectively predict ICIs efficacy (147). In recent years, studies have attempted to combine PET imaging with specific radiolabeled tracers to detect immune checkpoint proteins such as PD-L1 (148–150). PD-L1 expression levels can also be detected in CTCs; however, whether this indicator can serve as a predictive biomarker for ICIs efficacy remains unclear. Some studies have suggested that high PD-L1 expression in CTCs indicates a better prognosis (151–153); however, there are also results indicating no correlation between the two factors (154).

Genomic alterations can occur in tumor cells, and the TMB can be used to quantify somatic mutations in these cells. An increased TMB is linked to the production of additional neoantigens that can be identified by T cells. NSCLC is one of the tumor types with the highest TMB, suggesting that a high TMB is correlated with better immunotherapy efficacy, independent of PD-L1 expression levels. Importantly, less than 10% of nonsynonymous mutations induce immunogenicity, and currently, there is no definitive threshold for TMB to accurately predict the efficacy of ICIs, thereby limiting the clinical application of TMB as a biomarker (155). Nevertheless, the TMB combined with other predictive biomarkers (such as PD-L1 expression and the NLR) still has strong predictive power (156). The average copy number variation of chromosomal fragments reflects genomic instability and may serve as a potential surrogate marker for TMB. Specific types of genetic mutations in tumor cells are also linked to the efficacy of immunotherapy. A comprehensive analysis of transcriptional signals related to T-cell activation, antigen presentation, and the IFNγ pathway can also serve the purpose of predicting therapeutic efficacy. Currently, researchers are working to identify gene expression-based features that can accurately predict the efficacy of ICIs; however, the existing studies are in the early stages and require further investigation for clinical application (157).

Certain circulating biomarkers can also act as indicators predicting the effectiveness of immunotherapy. Circulating tumor DNA (ctDNA) consists of fragments released by tumor cells into the bloodstream, and ctDNA analysis can identify tumor-specific genetic alterations. The bTMB can be determined through an analysis of ctDNA, with a higher bTMB suggesting better outcomes following immunotherapy. Additionally, monitoring the dynamic changes in ctDNA levels can provide insights, as a decrease in ctDNA concentration during immunotherapy is associated with a favorable treatment response. Specific types of immune cells in the circulation are also linked to the efficacy of immunotherapy; for example, a decrease in the NLR may indicate better treatment outcomes. Other biomarkers, such as soluble PD-L1 (sPD-L1) and serum granzyme B, can also predict responses to immunotherapy to some extent. However, some studies have indicated that the levels of plasma sPD-L1 may not be significantly correlated with patient overall survival (158). Therefore, further exploration is needed. Peripheral blood biomarkers represent a significant advancement in clinical practice; however, owing to their relatively late development, their clinical utility still has certain limitations. The microbiome contributes to the development of innate and adaptive immunity and is linked to the effectiveness of immunotherapy. Studies have shown that specific types of microorganisms residing in the gut or lungs can predict the effectiveness of immunotherapy. However, the microbiome exhibits significant variability among individuals and is easily influenced by environmental factors, which limits its application in predicting the efficacy of immunotherapy. Future advancements in detection technologies are needed to determine the potential of the microbiome in personalized treatment.

TILs and TLSs reflect the immune status of the TME, while PD-L1 expression levels can indicate the immune evasion of tumor cells to some extent. The occurrence of mutations in tumor cells and the generation of neoantigens facilitate T-cell recognition and are crucial mechanisms for the efficacy of immunotherapy. Liquid biopsy technology can be used to detect substances released from tumor tissue into the circulation, allowing researchers to conveniently analyze specific characteristics of tumor tissue and monitor dynamic changes in real time. The microbiome can influence both the innate and adaptive immune responses of the host. The various predictive biomarkers for immunotherapy efficacy mentioned above affect antitumor immune responses through different mechanisms and can predict efficacy to some extent. However, the structural complexity of tumor tissues and their interactions with the host lead to challenges in predicting the efficacy of treatment based on a single biomarker. Currently, the only biomarker that has been approved and widely used for predicting the efficacy of immunotherapy in NSCLC is PD-L1. Nevertheless, as mentioned earlier, there are several issues with the use of PD-L1 alone to predict treatment efficacy. Other biomarkers, such as the TMB, have shown potential in predicting response in some clinical trials but have not yet been approved as standalone biomarkers for immunotherapy. A comprehensive analysis involving multiple biomarkers may better reveal the intrinsic characteristics of tumor tissues in different individuals. By leveraging new technologies such as machine learning and artificial intelligence, a holistic assessment of various biomarkers could yield improved predictive ability.

The widespread adoption of new technologies – particularly sequencing technologies – has led to a deeper understanding of biological predictive markers. However, a single marker often fails to provide ideal predictive value. The combined use of multiple markers may offer a better solution. By integrating different markers, researchers can capture disease mechanisms more comprehensively, leading to improved patient stratification and more effective treatment strategies. The application of multiple biomarkers represents an important advancement in personalized medicine, ensuring that treatment decisions are based on a more comprehensive and detailed understanding of each patient’s unique biological characteristics.

## 9 Conclusions

Precision medicine, as an important direction for the future development of healthcare, aims to provide individualized treatment plans for different patients. In the field of NSCLC, ICIs represent a significant breakthrough, making the in-depth study of biomarkers for predicting ICIs efficacy particularly important. Currently, research on biomarkers for ICIs efficacy mainly focuses on several aspects, including PD-L1 expression levels, tumor-infiltrating lymphocytes, TLS, Tumor genomic alterations, Transcriptome Signatures, circulating biomarkers, and microbiomes. However, there are still many limitations in current biomarker research. First, the detection methods for biomarkers have not yet been standardized, and different laboratories may use varying techniques and standards, leading to inconsistent results. Additionally, many studies primarily focus on the evaluation of single biomarkers, lacking comprehensive research on the combined analysis of multiple biomarkers, which may overlook complex biological mechanisms. Therefore, further research is urgently needed to clarify the true value of these biomarkers and explore their applicability in different patient populations. In future research, we should continue to explore biomarkers that can effectively distinguish ICIs responders from non-responders and promote the combined application of multiple biomarkers, as this will be a key direction for future development.
